# Editorial: Understanding the Interplay Between the Tumor Immune Microenvironment and Genetic Alterations in Thoracic Malignancies

**DOI:** 10.3389/fonc.2022.871544

**Published:** 2022-03-10

**Authors:** Giulia Pasello, Jordi Remon, Emanuela Felley-Bosco

**Affiliations:** ^1^ Department of Surgery, Oncology and Gastroenterology, University of Padua, Padua, Italy; ^2^ Medical Oncology 2, Istituto Oncologico Veneto Instituto di Ricerca e Cura a Carattere Scientifico (IOV IRCCS), Padua, Italy; ^3^ Department of Medical Oncology, Centro Integral Oncológico Clara Campal (HM-CIOCC), Hospital HM Nou Delfos, HM Hospitales, Barcelona, Spain; ^4^ Laboratory of Molecular Oncology, Department of Thoracic Surgery, University Hospital Zurich, Zurich, Switzerland

**Keywords:** tumor immune microenvironment, genetic alteration, lung cancer, mesothelioma, thymic epithelial tumor (TET)

Harnessing immune response to attack tumor cells has proven to be a successful treatment strategy against advanced thoracic malignancies. This immune checkpoint blockade (ICB) strategy with anti-PD-(L)1 and anti-CTLA4 monoclonal antibodies has reported durable responses and has significantly improved the overall survival rates either as monotherapy in selected tumors, or as a combination with immunotherapy and/or chemotherapy in all thoracic malignancies, except thymic epithelial tumors (TET) (1–3). PD-L1 expression in tumor cells is the most robust predictive biomarker for the efficacy of the ICB strategy. Nowadays, an increasing body of literature suggests a crucial role for the tumor microenvironment (TME) in cancer progression and therapeutic responses. Therefore, other potential biomarkers are being explored such as tumor-infiltrating lymphocytes (TILs), tumor-associated macrophages (TAMs), and cancer-associated fibroblasts (CAFs). Likewise, tumor mutational burden and immune-related genetic signatures are being tested with the aim to select those patients most likely to obtain a true benefit from this strategy, and avoid exposure to potential toxicity in patients who will not obtain clinical benefit (Chen et al.). In this Research Topic, a group of international authors discuss the current advances in the study of the interplay between the TME and genetic alterations in thoracic malignancies, such as non-small cell lung cancer (NSCLC), small cell lung cancer (SCLC), pleural mesothelioma (PM), and thymic epithelial tumors (TETs). This Frontiers in Oncology issue includes novel data (Alves et al.; Cao et al.; Chen et al.; Du et al.; Pezzuto et al.; Xu et al.) and review papers (Behrouzfar et al.; Grard et al.; Hiltbrunner et al.; Principe et al.; van Genugten et al. Wadowski et al.).

The predictive and prognostic role of the TME as a whole and the correlation of specific phenotypes with differential gene expressions and clinical-pathological features of lung adenocarcinoma has been recently investigated. The construction of a TME-score on the basis of the genetic signatures involved in T-cell activation, lymphocyte proliferation, and mononuclear cell proliferation has been suggested as a useful prognostic and predictive tool for patients receiving ICB (Chen et al.).

CD8+ T cells are one of the central effector cells in the immune microenvironment and play a vital role in the development and progression of lung adenocarcinoma (LUAD). Du et al. explored the key genes related to CD8+ T-cell infiltration in 529 LUAD-related samples from TCGA and developed a novel prognosis model based on these genes. The risk score was negatively related to CD8+ T-cell infiltration and correlated with the advanced tumor stage (Du et al.). Clinical applicability of this score could be relevant in the coming future as the adjuvant ICB strategy is accepted in completely resected PD-L1-positive stage II-IIIA NSCLC (4).

The crucial role of T-cell activation and TME modeling in predicting the survival of lung cancer patients has also been highlighted by the work by Cao et al., which showed longer survival in lung cancer patients functionally enriched with platelet endothelial cell adhesion molecule-1, a molecule involved in T-cell response regulation and migration (Cao et al.).

In the coming future it would be relevant to explore the role of potential biomarkers that select patients who cannot obtain benefit from the ICB strategy. As an example, in LUAD, the *STK11* and *KEAP1* mutations confer worse outcomes to immunotherapy among patients with KRAS mutant NSCLC but not among KRAS wild-type LUAD (5). Similarly, Grard et al. reviewed the role of homozygous co-deletion of type I interferons and CDKN2A, due to their co-localization in chromosome 9, in thoracic cancers and its consequences for therapy such as oncolytic therapy. Indeed, this co-deletion has been observed in a large proportion of mesothelioma patients and, together with the status of tumor suppressor BRCA-associated protein 1 (BAP1), is part of the genetic effect on the immune phenotype, as reviewed by Wadowski et al. In this and the complementary review by Hiltbrunner et al., they summarized the different studies documenting immune cells, differential infiltration, and the association with clinical outcome in mesothelioma. CDKN2A encodes for two proteins, p16/INK4A and p14/ARF, and Pezzuto et al. report that mesothelioma with strong immunoreactivity for p14/ARF has a high expression of ICB target PD-L1. It would be interesting to explore nuclear BAP1, which is used as a surrogate for wild-type function (6), in such a context. Indeed, in the TCGA study (7), the researchers found that type-I IFN signaling is associated with the status of BAP1.

Although mesothelioma is the sixth of the 31 most prevalent cancer types with a 38-interferon-stimulated genes signature (8), one aspect that is still underexplored is the priming for viral mimicry induction (9) which has been observed in an experimental model of mesothelioma development (10).

During the course of immunotherapy it will be important to follow immune responses longitudinally (11) and predict outcome, since it allows researchers to stratify patients into responders and non-responders. Principe et al. highlight the potential of doing so using pleural effusion, which is minimally invasive, and therefore easy to implement.

TETs are a heterogenous group of thoracic malignancies, mostly considered cold tumors except B3-thymoma and thymic carcinoma (12), reflecting a different TME according to the histologic subtype, which may negatively impact the tumor mutational burden, affecting ICB efficacy. Xu et al. report that *TP53* mutation is higher in hot TETs and correlates with worse prognosis compared with TETs without *TP53* mutations. Therefore, the genomic profile may have an influence in the immune sensitivity of TETs.

Alterations in microenvironmental metabolic characteristics are recognized as important means for cancer cells to interact with the infiltrating T cells within this TME (13). Molecular imaging has developed a wide array of tracers targeting metabolic pathways to understand metabolic reprogramming in cancer cells, as well as its effects on immune cells. van Genugten et al. provide an overview of currently available molecular imaging tracers for clinical studies and discuss their potential roles in the development of effective ICB strategies.

In the same way, the interconnection between metabolic pathways and immune response regulation has been suggested and a role of metabolic biomarkers as predictors of response to ICB is currently under investigation. Glycogen synthase kinase-3 (GSK3)-beta is a serine/threonine kinase involved in the phosphorylation of different components of the PI3K/AKT pathway as well as in PD-1/PD-L1 expression regulation and CD8+ T-cell activation. Positive expression of this biomarker in NSCLC samples showed a correlation with worse clinical stage and survival as well as with high PTEN but not with PD-L1 expression (Alves et al.).

Beyond genetic alterations in cancer cells, host genetics may influence thoracic cancer risk and pathogenesis and may shape TME features, thus representing a determinant predictor of treatment outcome.

Recent evidence has focused on genome-wide association studies (GWAS), which suggested a polygenic pattern of predisposition to lung cancer in some series (14). Likewise, single-nucleotide polymorphisms, somatic mutations, and epigenetic alterations are involved in TME refining and prediction of response to ICB. Lacking GWAS evidence on uncommon thoracic cancers such as PM and TET lead to *in vivo* models able to mimic human cancer development and finally to the identification of host genetic variants. Among these, the Cross Collaborative MexTAg mouse model offers a wide picture of host genetic make-up predisposing to the risk of asbestos-related mesothelioma and determining TME composition and the biological pathway involved in the immune response (Behrouzfar et al.).

In conclusion, evidence from original works and literature reviews collected within the present topic expands knowledge about the characterization of TME, its prognostic and predictive role in thoracic cancer malignancies, and finally deepens the relationship between the antitumor immune response and genetics of cancer and host. These findings may help clinicians to improve the risk-benefit ratio of treatment with ICB for patients with thoracic malignancies incorporating immune-related signatures in the future design of clinical trials.

## Author Contributions

GP: planned the Research Topic, invited coeditors and authors, edited and submitted papers, and finally wrote the editorial. JR and EF-B contributed to author invitation, paper editing and finally editorial writing. All authors contributed to the article and approved the submitted version.

## Conflict of Interest

The authors declare that the research was conducted in the absence of any commercial or financial relationships that could be construed as a potential conflict of interest.

## Publisher’s Note

All claims expressed in this article are solely those of the authors and do not necessarily represent those of their affiliated organizations, or those of the publisher, the editors and the reviewers. Any product that may be evaluated in this article, or claim that may be made by its manufacturer, is not guaranteed or endorsed by the publisher.

## References

[1] Remon J Aldea M Besse B Planchard D Reck M Giaccone G. Small Cell Lung Cancer: A Slightly Less Orphan Disease After Immunotherapy. Ann Oncol (2021) 32(6):698–709. doi: 10.1016/j.annonc.2021.02.025 33737119

[2] Reck M Remon J Hellmann MD. First-Line Immunotherapy for Non-Small-Cell Lung Cancer. J Clin Oncol (2022) 40(6):586–97. doi: 10.1200/JCO.21.01497 34985920

[3] Baas P Scherpereel A Nowak AK Fujimoto N Peters S Tsao AS. First-Line Nivolumab Plus Ipilimumab in Unresectable Malignant Pleural Mesothelioma (CheckMate 743): A Multicentre, Randomised, Open-Label, Phase 3 Trial. Lancet (2021) 397(10272):375–86. doi: 10.1016/S0140-6736(20)32714-8 33485464

[4] Felip E Altorki N Zhou C Csoszi T Vynnychenko I Goloborodko O. Adjuvant Atezolizumab After Adjuvant Chemotherapy in Resected Stage IB-IIIA Non-Small-Cell Lung Cancer (IMpower010): A Randomised, Multicentre, Open-Label, Phase 3 Trial. Lancet (2021) 398(10308):1344–57. doi: 10.1016/S0140-6736(21)02098-5 34555333

[5] Ricciuti B Arbour KC Lin JJ Vajdi A Vokes N Hong L. Diminished Efficacy of Programmed Death-(Ligand)1 Inhibition in STK11- and KEAP1-Mutant Lung Adenocarcinoma Is Affected by KRAS Mutation Status. J Thorac Oncol (2022) 17(3):399–410. doi: 10.1016/j.jtho.2021.01.532 34740862 PMC10980559

[6] Carbone M Adusumilli PS Alexander HR Jr. Baas P Bardelli F Bononi A. Mesothelioma: Scientific Clues for Prevention, Diagnosis, and Therapy. CA Cancer J Clin (2019) 69(5):402–29. doi: 10.3322/caac.21572 PMC8192079 31283845

[7] Hmeljak J Sanchez-Vega F Hoadley KA Shih J Stewart C Heiman DI. Integrative Molecular Characterization of Malignant Pleural Mesothelioma. Cancer Discov (2018) 8(12):1548–65. doi: 10.1158/2159-8290.CD-18-0804 PMC6310008 30322867

[8] Liu H Golji J Brodeur LK Chung FS Chen JT deBeaumont RS. Tumor-Derived IFN Triggers Chronic Pathway Agonism and Sensitivity to ADAR Loss. Nat Med (2019) 25(1):95–102. doi: 10.1038/s41591-018-0302-5 30559422

[9] Chen R Ishak CA De Carvalho DD. Endogenous Retroelements and the Viral Mimicry Response in Cancer Therapy and Cellular Homeostasis. Cancer Discov (2021) 11(11):2707–25. doi: 10.1158/2159-8290.CD-21-0506 34649957

[10] Sun S Frontini F Qi W Hariharan A Ronner M Wipplinger M. Endogenous Retrovirus Expression Activates Type-I Interferon Signaling in an Experimental Mouse Model of Mesothelioma Development. Cancer Lett (2021) 507:26–38. doi: 10.1016/j.canlet.2021.03.004 33713739

[11] Isaacs J Tan AC Hanks BA Wang X Owzar K Herndon JE 2nd. Clinical Trials With Biologic Primary Endpoints in Immuno-Oncology: Concepts and Usage. Clin Cancer Res (2022) 28(1):13–22. doi: 10.1158/1078-0432.CCR-21-1593 34312214 PMC8738124

[12] Yamamoto Y Iwahori K Funaki S Matsumoto M Hirata M Yoshida T. Immunotherapeutic Potential of CD4 and CD8 Single-Positive T Cells in Thymic Epithelial Tumors. Sci Rep (2020) 10(1):4064. doi: 10.1038/s41598-020-61053-8 32132638 PMC7055333

[13] Hanahan D. Hallmarks of Cancer: New Dimensions. Cancer Discov (2022) 12(1):31–46. doi: 10.1158/2159-8290.CD-21-1059 35022204

[14] Kachuri L Graff RE Smith-Byrne K Meyers TJ Rashkin SR Ziv E. Pan-Cancer Analysis Demonstrates That Integrating Polygenic Risk Scores With Modifiable Risk Factors Improves Risk Prediction. Nat Commun (2020) 11(1):6084. doi: 10.1038/s41467-020-19600-4 33247094 PMC7695829

